# Production of Biodegradable Polymeric Composites with the Addition of Waste

**DOI:** 10.3390/ma16186305

**Published:** 2023-09-20

**Authors:** Fernando Antonio da Silva Fernandes, Juan Carlos Valdés Serra, Dayriane do Socorro de Oliveira Costa, Camilo Andrés Guerrero Martin

**Affiliations:** 1 Department of Engineering (FAE), Campus Salinópolis, Federal University of Pará, Rua Raimundo Santana Cruz, S/N, Bairro São Tomé, Salinópolis 68721-000, PA, Brazil; 2 Laboratory of Composite Materials, Federal University of Tocantins, Quadra 109 Norte Ave. NS-15, ALCNO-14, Master Plan Norte, Palmas 77001-090, TO, Brazil; juancs@uft.edu.br; 3 Department of Engineering, Federal University of Rio de Janeiro, Rio de Janeiro 28999-999, RJ, Brazil; dayrianecosta@gmail.com; 4 Laboratório de Operações e Tecnologias Energéticas Aplicadas na Indústria do Petróleo, Faculty of Petroleum Engineering, Federal University of Pará, Salinópolis 68721-000, PA, Brazil; camiloguerrero@ufpa.br

**Keywords:** biopolymers, plastic, macaúba epicarp, biodegradable composite, technological properties, circular economy

## Abstract

Several solutions have been presented to minimize the environmental impact generated by polymers produced from petroleum resources. This work produced a biopolymer using glycerol, starch (<5) and macaúba epicarp fiber (10–15–20–25–30%) as reinforcement. The interaction of glycerol with starch was favored by the addition of acetic acid (CH_3_COOH). The pH was adjusted with sodium hydroxide (NaOH) at a concentration of 0.1 mol·L^−1^. The characterization was carried out through scanning electron microscopy (SEM), infrared reflectance—FTIR, water solubility, biodegradability and technological properties. Through the results obtained in this work, it is observed that the tensile strength and modulus of elasticity are influenced by the addition of the fiber concentration; the sample that received a 30% addition presented 19.17 MPa and 348.12 MPa, respectively. All samples showed low solubility in water and low density, in addition to a high rate of degradability in soil with mass loss corresponding to 59% over a period of three months. The results of this investigation are satisfactory for the production of materials that can be used in everyday life, replacing conventional plastic.

## 1. Introduction

The pollution generated by plastic and its impacts on the environment have attracted global attention from researchers, companies, governments and the general public [1,2]. Plastics produced from petroleum resources [3], which are only 110 years old [4], present a great threat to the environment because their demand has been growing for decades due to the ease of molding and transforming them into various products of daily use. Approximately, 52 kg of waste is discarded annually per person, including bottles, medical supplies, food packaging [3], plastic bags [5], clothing and building materials [6]. Electronic waste alone has an annual production rate of 3% to 4% in the world, and this value is estimated to increase about to 55 million tons per year by 2025 [7] and to 0.2 billion tons by 2060 [5]. In addition, the COVID-19 pandemic experienced by the world increased plastic consumption, especially disposables that were used as personal protective equipment [5]. The pollution generated by plastic nowadays is seen as a serious environmental problem, especially in the oceans, due to the slow physical and biological degradation and the result of floating microplastics, which are the most common pollutants in the aquatic environment, acting as a contaminant for all aquatic organisms [8].

In this context, research has been directed towards investigating the production of biocomposites with natural reinforcing fillers [9], also known as bioplastics, which may be a good option to replace conventional plastics [2,10]. Currently, the production of bioplastics is about 1% of the total production of conventional plastic [2]. Bioplastics are produced using biodegradable or compostable polymers, which are derived from natural sources or simply synthesized from substrates of natural origin [9], such as starch [2], natural oils, protein and cellulose [10]. Starch is a good raw material for the production of polymeric matrices due to its biodegradability and low cost, being easily processed in the presence of a plasticizer, in addition to having polar groups that interact with the hydroxyls of lignocellulosic fibers, thus resulting in a material with mechanical strength, in addition to its high amylose content favoring film formation [3,11,12]. As a way to improve the technological properties of bioplastics, a chemical modification is necessary with the addition of other biodegradable polymers together with starch [13]. An alternative that can improve the technological properties of this material is combining two or more materials in a biphasic form, with the continuous phase corresponding to the biodegradable polymeric matrix and the discontinuous phase consisting of the reinforcement [14]. One of these kinds of materials are natural fibers of vegetable/lignocellulosic origin, used as a reinforcement in a polymeric matrix, which result in materials with good mechanical properties that are suitable substitutes for synthetic fibers, such as glass and carbon fibers [15]. In general, the use of fibers produced from the addition of agroenergy residues as reinforcement in composites offers a low-cost and environmentally correct solution for the disposal of residues, in addition to the possibility of obtaining profits [16] and promoting the circular economy and bioeconomy that have been implemented as alternative models of economic production to encourage sustainable growth and development [17,18].

To find the results of mechanical tests, several researchers make comparisons using statistical techniques such as one-way analysis of variance (ANOVA), which was also used to identify the significant factor that contributed to the experimental conditions [19,20,21].

Among the various residues in the biodiesel production chain, we can highlight macaúba [22], which has a high production of residues resulting from the production of 4000 L of oil per hectare per year, being a high-quality oil with 73% saturated fatty acids and standing out as a raw material for biodiesel production in Brazil [23]. The fiber from the macaúba epicarp is a favorable option for improving the mechanical properties [24] of bioplastics because it can be incorporated into this material, improving its mechanical performance, offering high resistance, rigidity and low density, in addition to minimizing the problem related to the destination of this agroenergy residue [25]. The macaúba fruit is composed of the epicarp, mesocarp, endocarp and almond with 11, 23, 59 and 7% of the mass, respectively [26]. The epicarp appears thin, hard, brittle, fibrous and light brown; the mesocarp is yellow due to the presence of bioactive coloring compounds such as carotenoids (~200 µg/g) [27], mainly β-carotene [27]. The pulp of macaúba is also rich in lipids (~29%) [27], mainly fatty acids such as oleic (~53%), palmitic (~25%) and linoleic (~14%) [27] acids.

In this context, another widely used raw material is glycerol, which is the main by-product of biodiesel generation, with an average production of approximately 1 kg for every 9 kg of biodiesel, acting as a plasticizer and guaranteeing the polymeric matrix and better flexibility [28,29].

The literature presents several studies that show the potential of agroenergy residues and the importance of recycling, especially for the promotion of the regional circular economy [30]. This study evaluated the results of a biodegradable composite using natural and renewable resources, easily accessible and abundant, such as agroenergy residues that were successfully added to the matrix of biodegradable biopolymers. The results, in terms of mechanical properties, processability and degradability rate, meet the requirements of a high-quality product at an industrial level.

## 2. Materials and Methods

**Figure 1:**
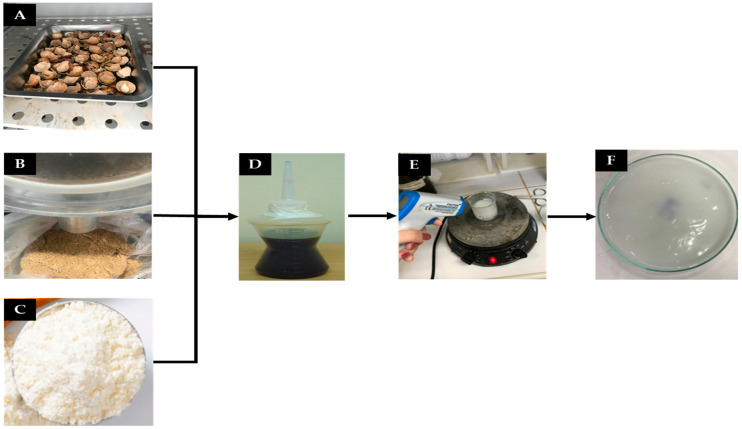
(**A**) Ground macaúba epicarp, (**B**) glycerol, (**C**) corn starch, (**D**) magnetic stirring procedure of the filmogenic solution with temperature control and (**E**) polymeric matrix in Petri dish before drying in an oven (**F**) Biopolymer sample.

The experimental flow of this investigation is illustrated in Figure 1.

### 2.1. Materials

Ripe macaúba fruits (Figure 1A) were collected and pulped in the rural area of the city of Aparecida do Rio Negro, Tocantins-Brazil (09°57′07″ S and 47°58′19″ W). The material was separated and packed in plastic bags to preserve its natural moisture. Industrialized corn starch was obtained from local businesses in the city of Palmas, Tocantins-Brazil (10°11′04″ S and 48°20′01″ W). P.A. glycerol, acetic acid and distilled water were provided by the Research Laboratory in Environmental Chemistry and Biofuels at the Federal University of Tocantins, LAPEQ-Palmas, Tocantins-Brazil (10°11′04″ S and 48°20′01″ W).

The macaúba epicarp fibers (Figure 1A) were separated and then dried in an electric oven with air circulation (Tecnal, São Paulo, SP, Brazil) for 24 h at the temperature of 60 °C, to remove residual water. Afterwards, the fibers were crushed (Figure 1B) in a knife mill (Solab, São Paulo, SP, Brazil) and classified using a 20 mesh sieve (1.33 mm). The grinding process reduced the porosity of the material, avoiding its negative influence on the mechanical strength of composites produced from natural fibers [31]. Starch (Figure 1C) was added, shortly after Glycerol (Figure 1D), then the sample was homogenized (Figure 1E), resulting in the investigated biopolymer sample (Figure 1F).

### 2.2. Characterization of Macaúba Epicarp Fiber

#### 2.2.1. Moisture Content

To determine the moisture content of the macaúba epicarp, 10 samples weighing 1 g (initial mass of the residue, Mi) were used, which were weighed on a digital precision scale (Marte, São Paulo, SP, Brazil). The samples were dried in an oven at the temperature of 105 °C for 4 h, then cooled in a desiccator for 15 min and weighed again. This process was repeated until reaching constant mass (final mass of the residue, Mf) (Instituto Adolfo Lutz). The determination of moisture content is expressed by Equation (1).

Mu = Mi − Mf(1)

where

Mu—mass of moisture lost during drying (g);
Mi—initial mass of the residue (g);
Mf—final mass of the residue (g).

The average moisture content is obtained through the arithmetic mean of the 10 analyzed samples, using Equation (2).

(2) $$u=\frac{Mu}{Mi}\times 100$$

where

u—residue moisture content (%);
Mu—mass of moisture lost during drying (g);
Mi—initial mass of the residue (g).

#### 2.2.2. Ash Content 

The determination of the ash content aims to identify the non-nitrogenated extract (NNE) and/or organic matter. In this process, the material is burned in an electric muffle (EDG, 1800, São Paulo-SP, Brazil) at the temperature (550~570 °C) for a previously determined period of time (Instituto Adolfo Lutz, 1985).

To determine the ash content, 10 samples containing 1 g of biomass were used. First, the empty porcelain crucibles were heated in a muffle until all the residues present were burned and then cooled in a desiccator. Subsequently, 1 g of biomass was added to each of the 10 crucibles (sample initial mass, Mi), then taken to the muffle at 550 °C for a period of 4 h and then cooled in a desiccator. After 1 h, the samples were weighed again (ash mass, Mf). The determination of ash content is calculated by Equation (3).

(3) $$T_{Cinzas}=\frac{100\times Mf}{Mi}\;$$

where

T_Cinzas_—ash content (%);
Mf—ash mass (g);
Mi—sample initial mass (g).

#### 2.2.3. Obtaining Polymer Matrices

The materials used in the production of the polymeric matrix are described in Table 1. This investigation was based on the casting technique [32]. The amount of raw material in each of the samples was previously defined, taking into account the mass that the Petri dish could support, as well as the temperature and time required for drying the samples. Therefore, the following formulations were prepared. 

To prepare the different samples described in Table 1, 50 mL of water was added to a 500 mL beaker, then heated with constant magnetic stirring until reaching the temperature of 50 °C. During heating, starch was added to the mixture, which continued to be heated to the temperature of 70 °C (close to the gelatinization temperature of starch). Then, acetic acid and glycerol were added to the mixture. Acetic acid (CH_3_COOH) acts by breaking glycerol bonds, facilitating its interaction with starch. Stirring was maintained up to the temperature range of 80~85 °C, obtaining a transparent mixture with gel-like consistency, in a slightly solid form.

The pH was maintained in a range between 6 and 8, by dripping a sodium hydroxide solution (NaOH) with a concentration of 0.1 mol L^−1^. The whole process took approximately 30 min. Soon thereafter, the samples were spread in the Petri dish (Figure 1A) and placed in an oven with air circulation (45 °C) for 12 h, and then remained at room temperature for 24 h. At the end, the specimens were molded to carry out the pre-tests of the mechanical tests. 

An important step during the placement of the samples (filmogenic solution) in the petri dish is maintaining the leveling of the samples, which is fundamental to avoid accentuated unevenness and differences in thickness (Figure 1F), as well as the correct temperature control during this process (Figure 1E), since a good consistency of the matrix after drying occurs at the optimal time for adding the reagents.

After carrying out a pre-test that evaluated the mechanical behavior of the samples, five formulations were investigated (Table 2).

For the production of the polymeric composites described in Table 2, the same production procedure used to prepare the matrices described in Table 1 was adopted. The crushed macaúba epicarp was added in the last stage of the production process. The sample was then maintained for another 10 min under stirring at a constant temperature of 80~85 °C. After that, the composite samples were placed in Petri dishes (Figure 1F) and kept at 45 °C for 12 h and then for 24 h at room temperature.

#### 2.2.4. Mechanical Properties

The mechanical tests were carried out with a Universal Testing Machine (EMIC, DL-3000) with a load cell of 5000 N, gripping speed of 4000 mm/min, based on the ASTM D638-08 Standard [33]. The specimens were made to measure 40 mm × 60 mm and were tested in relation to tensile strength, elongation at break and Young’s modulus. To evaluate the tensile strength of the film, Equation (4) was used.

(4) $$\sigma =\frac{F}{A}\;$$

where

σ—tensile strength (MPa);
F—last maximum breaking force (N);
A—cross-sectional area (mm).

For the rupture test, the ASTM D882-00 [34], 2001 recommendation was followed, according to Equation (5).

(5) $$A\left(\%\right)=\frac{Lf-Lo}{Lo}\times 100\;$$

where

A—elongation at break (MPa);
Lf—final elongation of the sample (mm);
Lo—initial sample size (mm).

Young’s modulus was determined using Equation (6).

(6) $$E=\frac{\sigma }{\varepsilon }$$

where

E—modulus of elasticity or Young’s modulus (MPa);
σ—tension (MPa);
ε—deformation (dimensionless);

Density was verified using cylindrical specimens measuring 2 cm in length × 2 cm in diameter, in triplicate, which were dehydrated in a desiccator containing silica gel for 21 days, according to Equation (7).

(7) $$d=\frac{m}{v}=\frac{m}{A_{E}\;}$$

where

m—sample mass (g);
v—sample volume (cm^3^);
A—sample area (cm^2^);
E—sample thickness (mm).

The solubility in water of filmogenic solutions was determined according to the method proposed by Gontard et al., 1994 [35]. The samples were prepared, in triplicate, in the form of 2 cm diameter discs. The sample’s initial mass was determined after drying for a period of 24 h in an oven at the temperature of 105 °C. After the first weighing, the samples were then immersed in a recipient containing 50 mL of distilled water and stirred for a period of 24 h. Then, the solubilized samples were removed from the water and dried again at the temperature of 105 °C for 24 h, to obtain the final mass.

#### 2.2.5. Scanning Electron Microscopy—SEM

For the scanning electron microscopy tests, samples measuring 1 cm × 1 cm were used, which were coated with a gold bath using Denton Vacuum Desk equipment. Samples were analyzed using (JSM-JOEL-SL–031, Tokyo, Japan) equipped with EDS Thermo Scientific NSS Spectral Imaging.

#### 2.2.6. Fourier Transform Infrared Spectroscopy—FTIR

The reflectance analysis was carried out using equipment with a universal diffuse reflectance accessory (UATR) and diamond crystal (ESPECTROFOTÔME-TRO NIR 900-PLS -Femto Indústria e Comércio de Instrumentos LTDA, São Paulo, Brazil). Through this technique, it was possible to identify the functional groups of polymeric composites. 

#### 2.2.7. Biodegradability

**Figure 2:**
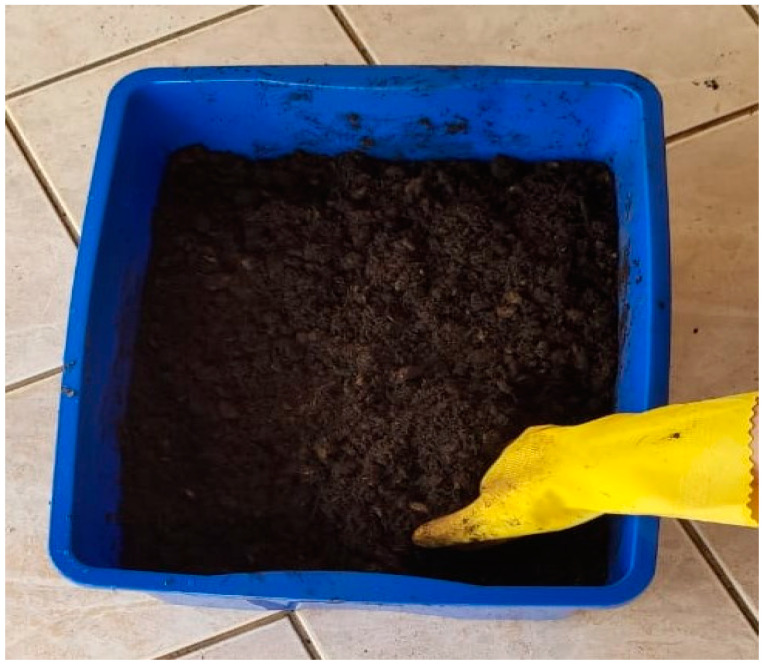
Substrate for biodegradation test.

The biodegradation test, by mass loss, was based on the ASTM G160-03 standard [36]. The composition of the substrate for the biodegradation test was 2 kg of dry horse manure, 2 kg of coarse sand, 2 kg of fertile soil with low clay content and 500 mL of water for homogenization, as shown in Figure 2.

The substrate was kept at rest for 90 days for maturation; during this period, humidity and pH tests were carried out, with reading performed monthly. The test was performed at room temperature and protected from sunlight. Analyses were performed in triplicate [37]. The mass loss was calculated using Equation (8).

PM = MI − MF(8)

where

PM—mass loss (g);
MI—initial mass (g);
MF—final mass (g).

The percentage of mass loss of the samples was calculated using Equation (9):

(9) $$PM(\%)=\frac{m-m^{\prime }}{m}\times 100$$

where

PM—mass loss (%);
m—initial mass (g);
m′—final mass (g).

#### 2.2.8. Experimental Design

For the experimental design, the software SISVAR Version 5.6 was used, through which it was possible to determine the analysis of variance (ANOVA). Then Tukey’s test was applied to determine the differences between the properties of the composites, where it was determined which means of treatment differed from each other, at the level of 5% probability (*p* < 0.05), which corresponds to 95% reliability. For these analyses, five treatments were considered for both polymer matrices and composites, with five repetitions for each analyzed factor (thickness, tensile strength, elongation at break and Young’s modulus). Thus, the results were presented by mean and standard deviation.

## 3. Results

**Figure 3:**
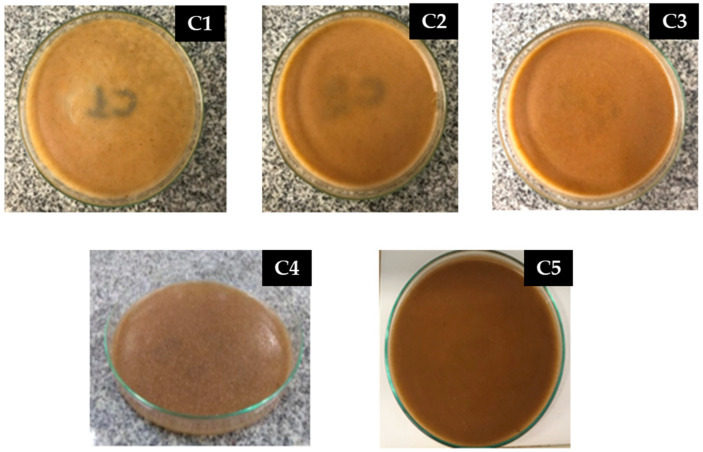
Polymer matrix before oven drying.

During the preparation of the samples, visual and tactile analyses were carried out to classify and guarantee that the films (Figure 3) were homogeneous, with a uniform color and did not present insoluble gelatin particles; in this way, the samples were flexible, with ease of handling, showing no ruptures or brittle areas. The thickness of the films was determined using a 0.05 mm precision caliper, with the thickness value being the arithmetic mean of five measurements taken in different parts of the samples.

### 3.1. Moisture Content of Macaúba Epicarp

The results obtained by analyzing the 10 macaúba epicarp samples (Table 3) for moisture content and ash content show that the average value obtained for moisture content is 4.2%, with a standard deviation of ±0.67%, with the lowest value corresponding to 3% and the highest to 4.97%. The ash content represents the inorganic matter of the macaúba epicarp fiber, and the average value obtained for this parameter is 4.6%, with a standard deviation corresponding to ±0.57%. The presence of high moisture content in plant fibers leads to a loss of mechanical properties in the composites due to the formation of bubbles during processing, which causes porosity in them [14,38]. All values in Table 3 are below the humidity value established for the application of lignocellulosic materials in composites, which, according to the literature is 5% [39,40].

### 3.2. Polymer Matrices

The results of the preliminary tests were important for the success of this investigation. It was verified that the polymer matrices are influenced by the addition of starch (<5 g), temperature (<85 °C) and drying time (30 min).

**Figure 4:**
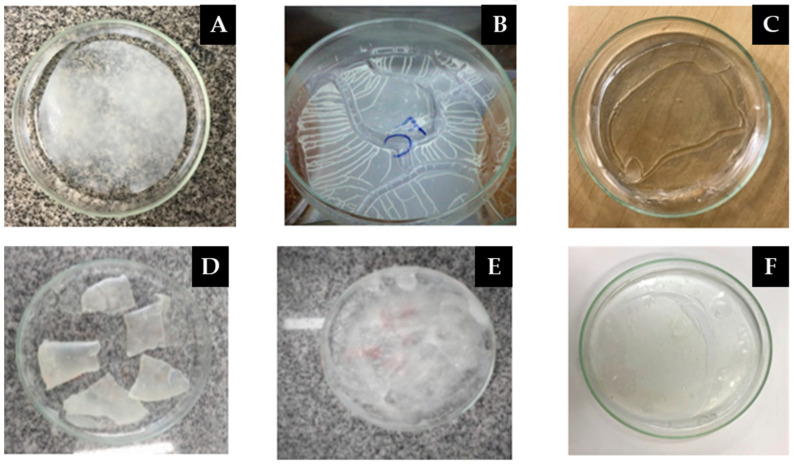
Visual presented of the Polymeric matrix tested in pre-test.

During these tests, it was observed that the excess of starch, time and temperature were not sufficient for the total drying of the matrix (Figure 4A). Subsequently, the film became brittle due to excess of acetic acid and drying time (Figure 4B).

The film that was dried for a longer time had a sticky appearance (Figure 4C), which then formed a more consistent polymeric matrix, but broke when drying (Figure 4D). Figure 4E presents the ideal consistency of the matrix before drying in an oven, whereas Figure 4F presents the film with good consistency when drying; this standard sample is investigated in this work.

### 3.3. Mechanical Properties 

**Figure 5:**
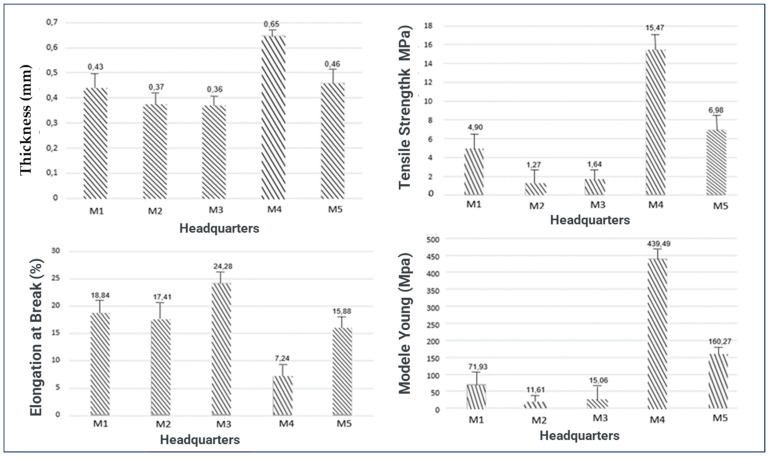
Polymeric matrix tested in pre-test.

The polymer matrices were submitted to mechanical tests. The experimental results of the mechanical tests and the average mean and standard deviation of the five specimens of each matrix (M1, M2, M3, M4 and M5) are presented in Table 4. Each matrix was analyzed in terms of thickness (mm), tensile strength (MPa), elongation at break (%) and modulus of elasticity or Young’s modulus (MPa); thus, the quality and strength of the polymer matrices produced can be analyzed. The averages of each factor analyzed in the mechanical tests are illustrated in Figure 5. The average and standard deviation referring to the mechanical properties of the investigated polymeric matrices are presented in Table 4.

The results illustrated in Figure 5 and the mean of the standard deviation referring to the mechanical properties of the investigated polymeric matrices presented in Table 4 show that the thicknesses in the matrices M1, M2 and M3 do not differ statistically from each other; it is emphasized that both received the same amount of starch. However, they differ statistically from the matrices with formulations M4 and M5, which received a higher amount of starch; therefore, the starch influenced the film thickness.

There was a variation of 0.38 mm~0.68 mm, and the greatest thickness obtained corresponded to the M4 matrix; the values found in this investigation are in agreement with the study by Zou et al. (2021) [41]. According to Gómez et al. (2020) [42], when producing a film using the casting technique, the thickness control largely depends on the viscosity of the film-forming solution. In very viscous solutions, they behave like Bingham fluids, that is, they do not flow under the agitation of their own weight; therefore, the solution must be spread with appropriate equipment, maintaining a known thickness on the support. In dilute solutions, thickness control occurs through the weight of the material obtained, requiring strict control of the shape of the support and the level of the oven, to avoid differences caused by unevenness during drying.

The tensile strength results show that there is a variation of 1.266 MPa~15.47 MPa, highlighting matrix M4 [43]. This value for the biodegradable film of the M4 formulation is close to that of the synthetic film, corroborating the potential of starch for this purpose. In general, tensile strength increased with starch concentration.

Elongation at break presented very representative results with ± 5% of significance, which can be explained by the variation of glycerol in the matrices. This elongation is in accordance with the ratio between the elongation of the specimen and its initial length; in other words, it measures the stretching capacity of the specimen [44]. The highest average was presented by M3 (24.28%), which also had the highest concentration of acetic acid in its composition.

The polymeric matrix M5 exhibited the lowest value, with an average equivalent to 7.24%, values according to the work of Kumar et al. (2021) [45]. In addition, ref. [45] obtained a variation of 6.86~33.39% in elongation at break, with the development of biocomposite films of corn starch polyvinyl alcohol; the results are in line with the results by Yurong and Dapeng (2020) [46], who obtained elongation values ranging from 5.89% to 57.4% when developing films composed of starch/PVA/glycerol.

The test result for Young’s modulus showed the tensile strength and elongation percentage, which indicates the stiffness of the film. The higher the Young’s modulus value, the stiffer the film. As it is directly linked to tensile strength, the matrix that obtained the highest resistance value also consequently obtained the highest value in Young’s modulus, highlighting the matrix of formulation M4 (439.49 MPa) [47]. In their study, Lim et al. (22021) [48] developed corn starch/PVA films with 452.08 MPa, this value being close to the value obtained in this work.

The results from the analysis of variance of the mechanical tests of the polymeric matrices obtained with corn starch and glycerol are presented in Table 5, in which it is possible to visualize the values of degree of freedom and mean square of the polymeric matrices.

### 3.4. Biopolymers

The results of the mechanical tests presented by the investigated composites, C1, C2, C3, C4 and C5, are illustrated in Table 4. These composites received reinforcement according to Table 2.

#### 3.4.1. Thickness

It is possible to verify in Table 6 that in increasing the percentage of reinforcement in the formulation, the thickness also increases (0.54 ± 0.65 mm), with no significant difference between treatments at the 5% level of significance.

According to Silva et al. (2020) [49], biopolymers were developed from macaúba epicarp flour and glycerol as a plasticizer, with thicknesses ranging from 0.175 mm to 0.87 mm. In a subsequent study by [41], composites containing plant fibers, including hemp, flax, ramie and jute, exhibited thicknesses from 0.47 mm to 1.27 mm. Notably, for composites with flax fiber, the thickness was recorded as 0.62 mm, corroborating the results obtained in the present study.

#### 3.4.2. Tensile Strength 

**Figure 6:**
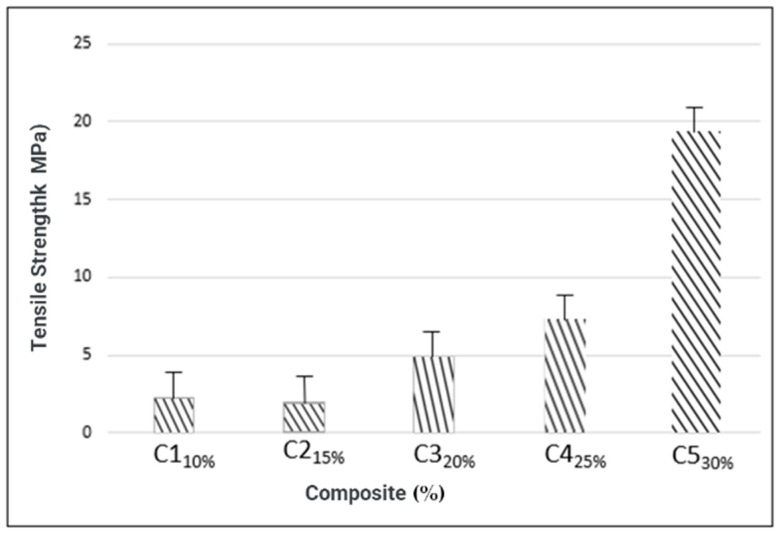
Tensile strength of composites.

The tensile strength of the composites (Figure 6) behaved similarly to the thicknesses; as the percentage of reinforcement increased, the strength also increased, except for composite C1 (2.259 MPa ± 0.41), which obtained a higher strength value than C2 (1.998 MPa ± 0.04). There was an increase of 1.99 MPa~19.17 MPa in the treatment tests, where the C5 composite had the highest tensile strength (19.168 MPa ± 0.77), followed by the C4 composites (7.286 MPa ± 2.09) and C3 (4.8764 MPa ± 0.16). Both are statistically different from each other, at the 5% probability level. These values are in agreement with studies by Gomes et al. (2019) [50], who produced biocomposites with coconut fiber and a matrix based on corn starch and glycerol that reached tensile strength values ranging from 1.64 MPa~25.7 MPa. Moura et al. (2021) [19], when developing biodegradable composites based on macaúba mesocarp flour with a matrix based on cassava starch, obtained values of 1.24 MPa~63 MPa, where they observed that as the fiber contents increased with slight variations, the tensile strength values also increased.

#### 3.4.3. Stretching Resistance (Rupture)

**Figure 7:**
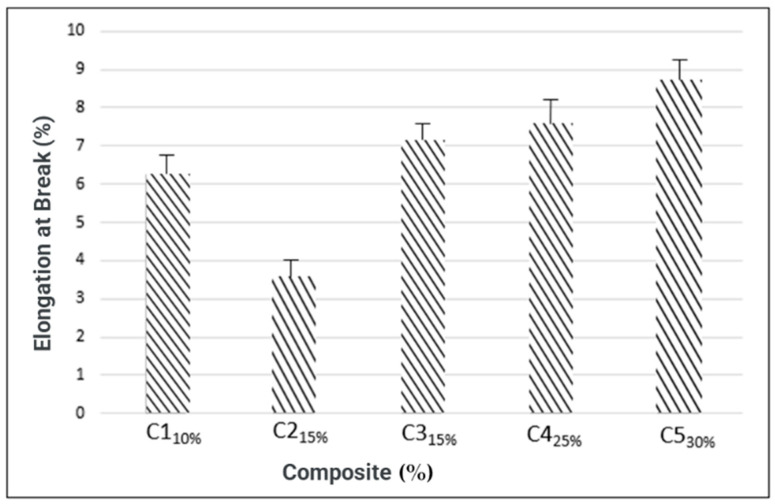
Resistance to elongation at break.

For resistance to elongation at break (Figure 7), only composite C2 differed statistically from the others (3.54%), while the highest value obtained (8.68%) corresponded to composite C5.

In studies by [19,50], biocomposites were developed with different matrices based on starch and natural fibers. In both cases, tensile strength values varied significantly, reaching up to 25.7 MPa and 63 MPa, respectively, as fiber contents increased. Previous work also demonstrated improvements in tensile strength by increasing the fiber percentage. The study by [32] used natural fibers from agro-industrial waste to create biocomposites, noting that increasing fiber content resulted in greater elongation at break. In the study by [50], composites reinforced with lignocellulosic fibers showed a 16% increase in tensile strength with the addition of 20% green coconut fiber. However, there was a 43% reduction in elongation at break, resulting in values of approximately 4.1%, similar to those obtained in this study with fiber concentrations corresponding to 15%. Ref. [51] produced biofilms incorporating cassava agro-industrial residues, resulting in a variation in Young’s modulus from 228.35 MPa to 708.11 MPa. The addition of 30% fiber resulted in elasticity of 311.67 MPa, corresponding to the values obtained in the C4 and C5 formulations of this study. Ref. [25] obtained composites reinforced with macaúba fibers, reaching values of 309 MPa to 488 MPa for fiber contents of 20% and 25%, respectively.

#### 3.4.4. Young’s Modulus

**Figure 8:**
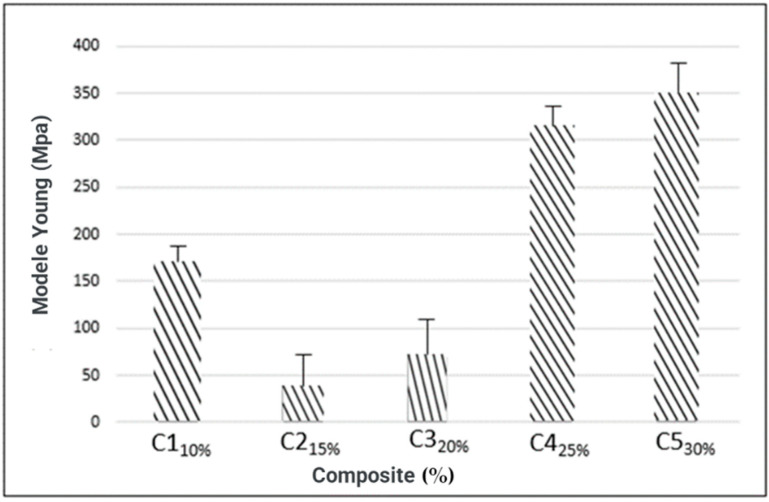
Modulus of elasticity/Young’s.

In Young’s modulus (Figure 8), there was a difference (5%) of significance between the factors. It was found that C4 and C5 stood out, presenting 310.7 MPa and 348.12 MPa, respectively, resulting from the highest percentage of reinforcement added (25%) and (30%), respectively [51]. The composite (C5) stands out with higher results of tensile strength and Young’s modulus. It is also noticeable that when tensile strength and modulus of elasticity increase, there is a decrease in elongation at break. The increase in the modulus of elasticity of composites is translated as an increase in the stiffness of the material; thus, the higher the modulus of elasticity, the stiffer the composite will be. Table 7 presents the variance analysis of the mechanical properties of biopolymer composites.

#### 3.4.5. Density

**Figure 9:**
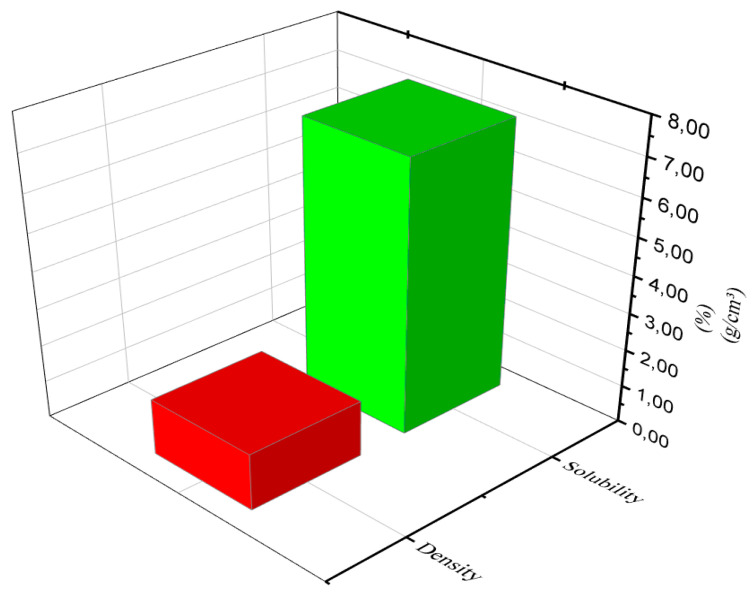
Density and water solubility of composite C5.

The density values of the composite C5: the best result, illustrated in Figure 9, indicated that there was reproducibility of the composite preparation process. Water solubility tests indicated a low value, which can be explained by the use of glycerol, which due to its hydrophilic character and the availability of hydroxyl groups, has a great influence on the solubility of starch films [52]. Glycerol interacts with the film matrix, making the free space between the chains larger, allowing water to enter and therefore providing solubility [47].

#### 3.4.6. Scanning Electron Microscopy (SEM)

**Figure 10:**
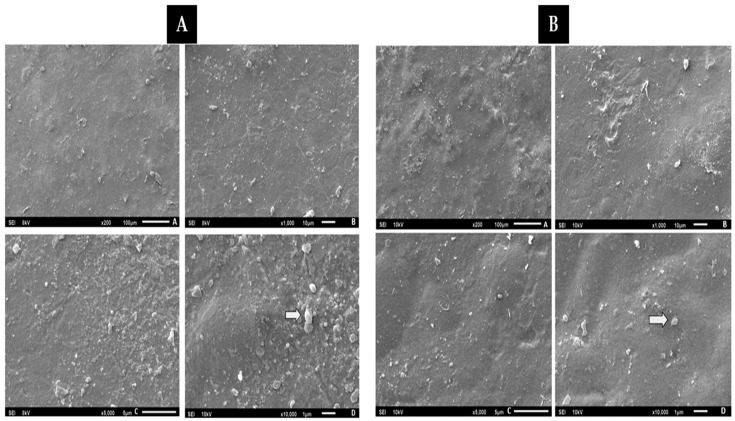
(**A**) Micrograph of C4 biopolymer—A (200×), B (1000×), C (5000×) and D (10,000×). (**B**) Micrograph of C5 biopolymer—A (200×), B (1000×), C (5000×) and D (10,000×).

Observing the SEM images of composite C4 shown in Figure 10A,B, which also illustrates composite C5, it is possible to identify the presence of ungelatinized starch granules (arrows) and epicarp fibers that did not adhere completely to the polymeric matrix. The two composites present images with a rough appearance, with depressions, and homogeneous zones. Analyzing the cross-section of the film, an irregular structure is observed, with reliefs, grooves and granules of ungelatinized starch and fibers. The presence of sulcus incurs due to the presence of microbubbles formed during the gelatinization process [53]. The presence of intact starch granules reduces flexibility and elongation, resulting in a less cohesive matrix. However, it also shows a homogeneous surface, without cracks, with a granular, porous structure and some surface irregularities. These results are concurrent with work carried out by [49].

Cross-sectional analysis of the films revealed an irregular structure with grooves, ungelatinized starch granules and fibers. The formation of grooves is attributed to the presence of microbubbles generated during gelatinization. The high flour/fiber concentration of the macaúba epicarp made the film more porous, which could lead to phase separation (Zimmermann et al., 2014 [53]). These findings are congruent with previous results, such as those by [49], who observed a leveled and continuous surface in macaúba mesocarp flour films with glycerol and clove oil as plasticizers, although they still had some porosity. Other studies, such as Maniglia and Tapia-Blácido (2016), who developed biopolymers based on babassu coconut mesocarp starch, identified predominantly smooth granules, but some areas exhibited granular surfaces due to proteins, lipids and fibers present. Micrographs also showed rough structures adhered to the surface, such as layers of lignin, hemicellulose and waxes, typical characteristics of this material. 

#### 3.4.7. Spectroscopy (FTIR)

**Figure 11:**
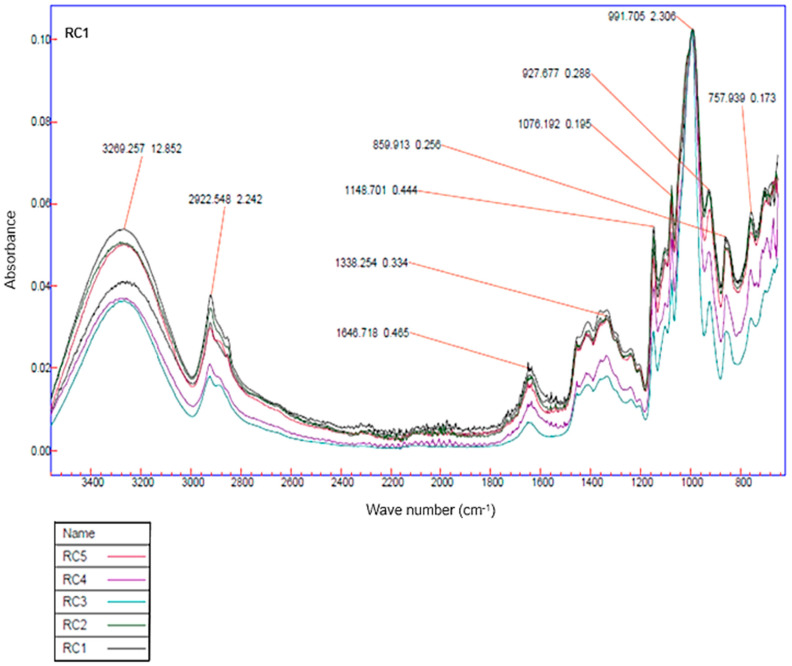
FTIR spectroscopy of biodegradable composites.

The illustrated spectroscopy (Figure 11) shows that the composites presented similar behavior, with small differences in the results of the bands; thus, all treatments had a standardized infrared absorbance behavior. The most pronounced peaks were approximately 3269.25 cm^−1^ for the composite (C2), characteristic of the NH_2_ bond, caused by Nitrogen bridges, followed by 2922.57 cm^−1^ (C5), which corresponds to the elongation of the CH_2_ bond and 991.705 cm^−1^ (C1), referring to the CO bridge, corresponding to the amide group with the protein group [26]. In line with this, peaks in the bands from 1150 to 1014 cm^−1^ are observed in corn starch films containing lignocellulosic fibers, as in the study by [49], who incorporated fibers from the trunk of the macaúba palm tree into composites. This increase in peak intensity can be attributed to the addition of glycerol during bioplastic synthesis [10]. Results similar to those of this study were observed in [13]), which produced biodegradable composites with a pea starch matrix and macaúba fibers. The broad absorption band corresponding to the O-H stretching was evidenced in the spectra, indicating hydrogen bonding interactions between the composite components during processing. In addition, bands close to 1600–1640 cm^−1^ were identified, related to the angular fold of the O-H in water molecules, indicating interactions between the components of the formulations, including starch, glycerol and fiber.

#### 3.4.8. Biodegradability

The result presented using the biodegradability test of the investigated samples is in accordance with ASTM G160-03 Standard [36]. The samples investigated in the period of 90 days kept at room temperature (~32.5 °C) showed pH 7.3, soil with moisture (~19.6%), slightly less than the value indicated by the standard.

The mean and standard deviation of the sample mass (C5) is shown in Table 8 for the biodegradation test.

It is observed that as the time in contact with the soil increases, the amount of mass loss of the composite increases. According to Siracusa (2019), the principal change that a degradable polymer undergoes is the reduction in molecular weight over a certain period of time. In the present study, the treatment showed a loss of mass equivalent to 40.40%.

## 4. Conclusions

The production of bioplastic with the addition of glycerol, starch and crushed macaúba fiber, investigated in this research, resulted in a material that can replace conventional plastics in everyday life, mainly bags. Technological properties presented as tensile strength (1.99 MPa to 19.17 MPa), resistance to elongation at break statistically presented (8.68%) and Young’s modulus (37.08 MPa to 348.12 MPa) favor the production of this material. In addition to replacing conventional plastic as packaging, the material produced encourages the circular economy with macaúba cultivation and correctly disposes of glycerol, a biodiesel residue.

As a future study for further research, the following is suggested:

- Replacement of glycerol by fatty acid, a residue from the biodiesel production chain;
- Since the macaúba epicarp fiber acted as a good reinforcement, promoting an improvement in the mechanical properties of the composite, an investigation with concentrations greater than 30% is therefore suggested to evaluate the influence on the technological properties;
- High concentrations of glycerol and acetic acid originate fragile and gelatinous polymeric matrices, so a new reagent that acts with glycerol must be investigated to favor its use;
- More research is underway to understand the chemical and physical changes that directly influence biopolymer characteristics and properties (mechanical, physical).

## Figures and Tables

**Table 1:** Investigated formulations for the production of polymeric matrices.

Matrix	Corn Starch (g)	Water (mL)	Glycerol (mL)	Acetic Acid (mL)
M1 M2 M3 M4 M5	3.00 3.00 3.00 5.00 5.00	50.00	1.00 3.00 1.00 1.00 2.00	1.00 1.00 2.00 1.00 1.00

**Table 2:** Percentage of the matrix sample and macaúba epicarp fiber for the production of polymeric composites.

Composite	Matrix Sample (%)	Macaúba Epicarp Fiber (%)
C1 C2 C3 C4 C5	90 85 80 75 70	10 15 20 25 30

**Table 3:** Moisture percentage of macaúba epicarp fiber.

Sample	Moisture (%)	Ash Content (%)
1 2 3 4 5 6 7 8 9 10	3.31 4.43 4.89 3.88 4.97 4.30 4.77 3.79 3.00 4.51	5.12 4.48 4.74 3.89 5.02 5.66 4.23 3.98 4.09 4.78
Average mean	4.20	4.60
Standard deviation	±0.67	±0.57

**Table 4:** Average mean and standard deviation referring to the mechanical properties of the polymer matrices.

Matrix	Thickness (mm)	Tensile Strength (MPa)	Elongation at Break (%)	Young’s Modulus (MPa)
M1 M2 M3 M4 M5	0.43 ± 0.02 0.37 ± 0.02 0.36 ± 0.02 0.65 ± 0.03 0.46 ± 0.04	4.90 ± 0.41 1.266 ± 0.04 1.645 ± 0.16 15.470 ± 2.09 6.977 ± 0.77	18.839 ± 3.9 17.414 ± 1.69 24.278 ± 1.04 7.24 ± 0.62 15.882 ± 2.12	71.934 ± 1.16 11.614 ± 0.11 15.06 ± 1.52 439.49 ± 3.20 160.27 ± 4.73

Note: Média das amostras do not differ statistically from each other, using Tukey’s test at the 5% probability level (*p* ≤ 0.05).

**Table 5:** ANOVA of mechanical tests of matrices.

VS	DF	SM			
		Thickness (mm)	Tensile Strength (MPa)	Elongation at Break (MPa)	Young’s Modulus (MPa)
Matrix	4	0.053	151.07	401.84	150.56
Error	21	0.002	0.882	4.153	190.06
Total	25	-	-	-	-
CV (%)	-	14.17	18.56	11.40	12.10

Note: VS: variation source; DF: degree of freedom; MS: mean square; CV: coefficient of variation.

**Table 6:** Mean and standard deviation referring to the mechanical properties of the composites.

Composite	Thickness (mm)	Tensile Strength (MPa)	Elongation at Break (%)	Young’s Modulus (MPa)
C1 C2 C3 C4 C5	0.54 ± 0.04 0.58 ± 0.04 0.60 ± 0.02 0.62 ± 0.04 0.65 ± 0.12	2.259 ± 0.41 1.998 ± 0.04 4.8764 ± 0.16 7.286 ± 2.09 19.168 ± 0.77	6.238 ± 3.9 3.545 ± 1.69 7.127 ± 1.04 7.567 ± 0.62 8.679 ± 2.12	168.07 ± 15.08 37.08 ± 1.11 68.76 ± 3.52 310.7 ± 37.4 348.12 ± 13.73

Note: In the columns, means not differ statistically from each other, using Tukey’s test at the 5% probability level (*p* ≤ 0.05).

**Table 7:** ANOVA of the mechanical tests of the composites.

VS	DF	MS			
		Thickness (mm)	Tensile Strength (MPa)	Elongation at Break (MPa)	Young’s Modulus (MPa)
Composite	4	0.007	187.61	13.45	939.23
Error	21	0.002	0.56	1.22	845.23
Total	25	-	-	-	-
CV (%)	-	11.28	17.78	17.62	19.34

Note: VS: variation source; DF: degree of freedom; MS: mean square; CV: coefficient of variation.

**Table 8:** For the biodegradation test.

Time (days)	Description	
	Average Weight (g)	Mass Loss (%)
0 30 60 90	0.52 ± 0.04 0.48 ± 0.12 0.39 ± 0.03 0.31 ± 0.06	- 7.69 ± 0.18 18.75 ± 0.21 20.51 ± 0.27
Total	0.21 ± 0.03	40.38 ± 0.26

## Data Availability

Data available on request.
